# Developing a Phosphodiesterase 10A Inhibitor as a Novel Therapeutic Agent for Triple-Negative Breast Cancer

**DOI:** 10.3390/cells15151374

**Published:** 2026-07-30

**Authors:** Mrityunjoy Biswas, Md Manirujjaman, Jovanny Zabaleta, Dorota Wyczechowska, Jone Garai, Qingzhao Yu, Luis Del Valle, Samarpan Majumder, Timothy Kayes, Xi Chen, Adam B. Keeton, Lucio Miele, Yulia Y. Maxuitenko, Nan Li, Gary A. Piazza, Fokhrul Hossain

**Affiliations:** 1 Department of Genetics, Louisiana State University Health Sciences Center-New Orleans (LSUHSC-NO), New Orleans, LA 70112, USA; mbisw2@lsuhsc.edu (M.B.); mmanir@lsuhsc.edu (M.M.); smaju1@lsuhsc.edu (S.M.); lmiele@lsuhsc.edu (L.M.); 2 Department of Interdisciplinary Oncology, Louisiana State University Health Sciences Center-New Orleans (LSUHSC-NO), New Orleans, LA 70112, USA; jzabal@lsuhsc.edu (J.Z.); dwycze@lsuhsc.edu (D.W.); jgarai@lsuhsc.edu (J.G.); ldelva@lsuhsc.edu (L.D.V.); tkayes@lsuhsc.edu (T.K.); 3 School of Public Health, Louisiana State University Health Sciences Center-New Orleans (LSUHSC-NO), New Orleans, LA 70112, USA; qyu@lsuhsc.edu; 4 Department of Pathology, Louisiana State University Health Sciences Center-New Orleans (LSUHSC-NO), New Orleans, LA 70112, USA; 5 Department of Drug Discovery & Development, Harrison College of Pharmacy, Auburn University, Auburn, AL 36849, USA; xzc0059@auburn.edu (X.C.); abk0039@auburn.edu (A.B.K.); yym0001@auburn.edu (Y.Y.M.); gap0034@auburn.edu (G.A.P.); 6 LSU-LCMC Health Cancer Center, New Orleans, LA 70112, USA; 7 Department of Biochemistry and Molecular Genetics, The University of Alabama at Birmingham, Birmingham, AL 35294, USA; sunlinan.82@gmail.com

**Keywords:** TNBC, ADT-030, PDE10, mammospheres, paclitaxel

## Abstract

**Highlights:**

**What are the main findings?**

- Identified phosphodiesterase 10A as a potential novel therapeutic target for triple-negative breast cancer.
- Demonstrated that the novel phosphodiesterase 10A inhibitor, ADT-030, exhibits antitumor activity and enhances chemotherapeutic efficacy in preclinical models of triple-negative breast cancer.

**What are the implications of the main findings?**

- The results showed that phosphodiesterase 10A is a candidate therapeutic target and that ADT-030 represents an attractive drug development candidate for triple-negative breast cancer.
- Further study of the role of phosphodiesterase 10A in triple-negative breast cancer and the mechanism of action of ADT-030 is warranted to support further translational research to advance ADT-030 or related compounds to clinical trials.

**Abstract:**

Triple-negative breast cancer (TNBC) is a highly aggressive subtype of breast cancer with limited therapeutic options for patients at high risk of disease recurrence and metastasis. The cyclic nucleotide-degrading enzyme, phosphodiesterase 10A (PDE10), that hydrolyzes both cAMP and cGMP has been previously reported to be expressed in multiple cancers and regulates key cellular signaling pathways involved in cancer cell proliferation, survival, and maintenance of stem cell-like properties. We found that PDE10 overexpression was associated with poor relapse-free survival of TNBC patients and identified its potential as a therapeutic target for TNBC using a novel inhibitor, ADT-030. Our results showed that ADT-030 inhibited the growth of TNBC cells, reduced colony-forming efficiency and enhanced the therapeutic efficacy of paclitaxel. A TNBC mouse model demonstrated that oral administration of ADT-030 significantly suppressed syngeneic tumor growth and enhanced the antitumor efficacy of paclitaxel. ADT-030 treatment altered differentially expressed genes (DEGs), signaling pathways, and cellular processes. Overall, our findings suggest that ADT-030, as a monotherapy or in combination with standard-of-care chemotherapy, may be an effective therapeutic approach for TNBC. Further studies are warranted to better understand the oncogenic role of PDE10 in TNBC and the mechanisms by which ADT-030 modulates the tumor microenvironment (TME) and enhances chemotherapy response.

## 1. Introduction

Triple-negative breast cancer (TNBC) is a highly aggressive form of breast cancer, accounting for 15–20% of the disease [1,2,3]. TNBC is characterized by the lack (or very low levels) of immunohistochemically detectable estrogen receptor (ER), progesterone receptor (PR), and the lack of amplification of human epidermal growth factor receptor 2 (HER2) [4]. Due to the lack of these therapeutic targets, TNBC patients do not respond to hormonal or HER2-targeted therapies [4] and are prone to disease recurrence and metastasis [5]. Treatment strategies for TNBC primarily rely on surgery, chemotherapy, and radiation therapy [6,7]. The therapeutic landscape of TNBC has expanded to include immunotherapies and PARP inhibitors in certain cases [8,9]. Unfortunately, the effectiveness of chemotherapy for TNBC is undermined by debilitating side effects and rapid emergence of chemo-resistant clones [10,11]. Despite recent advances, TNBC still has low survival rates, highlighting the need for more effective treatments [12]. Therefore, it is essential to identify novel molecular targets and inhibitors to improve survival for patients diagnosed with TNBC.

Phosphodiesterase 10A (PDE10), a cyclic nucleotide-degrading enzyme that hydrolyzes both cyclic adenosine monophosphate (cAMP) and cyclic guanosine monophosphate (cGMP), has been identified as a potential oncoprotein and therapeutic target which is overexpressed in several tumors, including colon, lung, and ovarian cancers, where it promotes cancer cell proliferation and survival by suppressing cAMP/PKA and cGMP/PKG signaling [13,14,15]. Known PDE10 inhibitors and genetic knockdown of *Pde10a* were reported to selectively inhibit colon, lung, and ovarian cancer cell proliferation and induce apoptosis. PDE10 inhibitors were also shown to impair migration and invasion in preclinical models, and sensitize tumors to chemotherapy, while mitigating doxorubicin-induced cardiotoxicity [15,16]. Notably, PDE10 inhibition by small molecules results in dual suppression of key oncogenic driver pathways, Wnt/β-catenin and RAS-mediated MAPK/AKT [13,14,15]. These studies suggest that targeting PDE10 opens a new strategy for treating TNBC. However, the role of PDE10 in TNBC has not been well studied and currently available PDE10 inhibitors that were developed for CNS disorders such as schizophrenia and Huntington’s disease are not suitable for cancer indications due to low systemic bioavailability and side effects including sedation.

Piazza and his colleagues have developed and characterized a novel PDE10 inhibitor, ADT-030 [17,18]. In a recent study, Bandi et al. demonstrated that ADT-030 is a potent antitumor agent for pancreatic ductal adenocarcinoma (PDAC) that acts by a mechanism consistent with known PDE10 inhibitors by increasing cAMP/cGMP levels and activating PKA/PKG to suppress Wnt/β-catenin and RAS-mediated MAPK/AKT signaling [18]. Oral administration of ADT-030 significantly suppressed tumor growth in mouse models of PDAC without apparent toxicity. Moreover, ADT-030 potentiated the chemotherapeutic activity of gemcitabine plus nab-paclitaxel, a standard-of-care treatment for PDAC [18]. In another study, Gazi et al. reported durable and robust antitumor activity of ADT-030 in multiple mouse tumor models [17]. Additionally, ADT-030-mediated tumor growth inhibition was found to be in part immune-dependent, as ADT-030 treatment enhanced anti-PD-1 therapy [17]. Both studies reported that ADT-030 treatment reduced metastasis and significantly extended survival in mouse tumor models [17,18]. In this study, through Kaplan–Meier analysis, we find that PDE10 overexpression in TNBC is associated with poor survival. Results from our in vitro experiments show that ADT-030 significantly inhibited TNBC cell growth, mammosphere growth, and colony-forming ability. Our in vivo studies showed that ADT-030 suppressed syngeneic TNBC tumor growth and had an additive anticancer effect when combined with standard-of-care chemotherapy for TNBC, paclitaxel. Overall, our findings suggest that ADT-030 as a monotherapy or in combination with chemotherapy may offer a novel and effective therapeutic strategy for TNBC.

## 2. Methods and Materials

### 2.1. Cell Culture

The human TNBC cells MDA-MB-231, MDA-MB-468, and mouse TNBC cells C0321 were cultured and maintained in Gibco™ DMEM, (Gibco, Waltham, MA, USA), supplemented with 10% fetal bovine serum, (R & D System, Minneapolis, MN, USA) 100 IU/mL of penicillin, and 100 µg/mL of streptomycin medium. For mammospheres generation, cells were cultured in MammoCult™ Basal Medium (STEMCELL Technologies, Vancouver, BC, Canada), supplemented with 10% MammoCult Supplement, 0.4% heparin, 0.5% hydrocortisone, 150 IU/mL of penicillin, and 150 µg/mL of streptomycin. The cells were cultured in humidified incubators (Eppendorf, Framingham, MA, USA) in 5% CO_2_ at 37 °C.

### 2.2. Reagents and Antibodies

The compound ADT-030 was synthesized as described earlier; briefly, ADT-030 was synthesized as (Z,S)-2-(5-methoxy-2-methyl-1-(3,4,5-trimethoxy-benzylidene)-1H-inden-3-yl)-N-(1-methylpyrrolidin-3-yl) acetamide, based on a procedure originally described in U.S. patent 20200223815 [19], using 3-(4-methoxyphenyl)-2-methylacrylic acid as the starting material [17]. MammoCult™ Basal Medium (Human) (Cat. #05621), MammoCult supplement (Cat. #05622), heparin (Cat. #07980), and hydrocortisone (Cat. #07925) were purchased from STEMCELL Technologies, (Vancouver, BC, Canada). Rabbit monoclonal anti-PDE10A (AB227829, Abcam, Waltham, MA, USA) and mouse anti-β-actin (sc69879, Santa Cruz Biotechnology, Santa Cruz, CA, USA) were used for Western blot analysis. Fluorochrome-conjugated antibodies, CD3 (Cat. #562600, CD4 (Cat. #553651), CD8a (Cat. #553035), CD11c (Cat. #553802), CD11b (Cat. #571486), Gr1 (Cat. #563849), Ly6C (Cat. #571441), Ly6G (Cat. #571207), and F4/80 (Cat. #569224) were obtained from BD Biosciences (Franklin Lakes, NJ, USA), and CD45 (Cat. #56-0451-82) was obtained from eBioscience, (San Diego, CA, USA).

### 2.3. Western Blotting

After treatment with ADT-030, cells or mammospheres were collected and lysed in RIPA buffer containing 1 mM PMSF, 1 mM sodium orthovanadate (Santa Cruz Biotechnology, Santa Cruz, CA, USA), and 1 mM Protease and Phosphatase Inhibitor Cocktail (Thermo Scientific, Waltham, MA, USA). Protein concentrations were measured by Pierce^TM^ BCA Protein Assay kits (Thermo Scientific, Waltham, MA, USA). Lysates were separated by SDS–polyacrylamide gel electrophoresis under reducing conditions and then transferred onto polyvinylidene difluoride membranes (Immobilon^R^-FL; Merck Millipore, Tullagreen, Carrigtwohill, Ireland). The membrane was blocked with 5% skim milk for 1h at room temperature (RT). Membranes were incubated with rabbit monoclonal anti-PDE10A (AB227829, Abcam, Waltham, MA, USA) overnight at 4 °C and mouse monoclonal anti-β-actin (sc69879, Santa Cruz Biotechnology, Sant Cruz, CA, USA) for 1h at RT. It was then incubated with HRP-conjugated anti-rabbit IgG or anti-mouse IgG for 1h at RT. Specific bands were analyzed using Immobilon^R^ Forte Western HRP substrate (EMD Millipore, Burlington, MA, USA) and Chemidoc instrument (Bio-Rad, Hercules, CA, USA).

### 2.4. Cell Viability, Colony Forming, and Cytotoxicity Assays

Human MDA-MB-231, MDA-MB-468, MCF10A, and mouse C0321 cells were treated with 0, 0.01, 0.1, 0.5, 1, 10, and 50 µM of ADT-030 for 48 h. Cell viability was determined by MTT assay (ATCC, Manassas, VA, USA) according to the manufacturer’s instructions. For colony-forming assay, cells were treated once with vehicle or 10, 100, 500 nM, 1, and 5 µM of ADT-030, cultured for fourteen days, and stained with crystal violet. Colonies were imaged to analyze the effect of ADT-030 on colony formation. To analyze the toxicity of ADT-030 on immune cells, female FVB mice were treated daily with vehicle or ADT-030 (50 mg/kg body weight, orally) for seven days. After seven days of treatment, spleen and blood were collected. Spleen was processed to isolate spleen cells, and the cells were stained with fluorescence-conjugated antibodies for 1h. Blood (100 µL) was stained with fluorescence-labeled antibodies for 1h and lysed with RBC lysis buffer. Cells were washed with PBS and analyzed by flow cytometry (BD FACSymphony™ A3, BD Bioscience, San Jose, CA, USA).

### 2.5. Mammosphere Assay

MDA-MB-231 and MDA-MB-468 cells were plated on ultra-low attachment 24-well plate (Corning Life Sciences, Tewksbury, MA, USA) using MammoCult Human Medium Kit (STEMCELL Technologies, Vancouver, BC, Canada) for mammospheres formation. Seven days later, mammospheres (P0) were collected by gentle centrifugation and briefly trypsinized to obtain a single-cell suspension. Cells (1 × 10^4^) were plated on an ultra-low attachment 6-well plate (Corning Life Sciences, Tewksbury, MA, USA) with complete MammoCult growth medium. Cells were treated with vehicle (DMSO) or different concentrations of ADT-030 (1 and 5 µM) alone or in combination with paclitaxel (2 nM). After seven days of treatment, mammospheres were imaged and counted. For Western blot (WB) analysis, P1 mammospheres were cultured for 7 and 14 days, and then collected and processed for WB analysis.

### 2.6. T Cell Proliferation Assay

Bone marrow-derived myeloid-derived suppressor cells (BM-MDSCs) were generated from FVB mice as described previously [20]. Briefly, bone marrow cells were collected from FVB mouse femur and tibia bones and cultured with G-CSF, GM-CSF, and IL-6 (20 ng/mL each) for four days to generate BM-MDSCs in the presence or absence of ADT-030 (1, 5, 10, or 50 µM). T cell proliferation was measured using carboxyfluorescein succinimidyl ester (CFSE) dye dilution using flow cytometry, as described [20,21]. Briefly, T cells were isolated from naïve FVB mouse spleen using a T cell (CD3) isolation kit (STEMCELL Technologies, Vancouver, BC, Canada). Isolated T cells were then labeled with 1 μM CFSE. CFSE-labeled T cells were co-cultured with BM-MDSCs at a 4:1 (T cells: MDSC) ratio in a 24-well culture plate coated with anti-CD3 and anti-CD28 (1 µg/mL). T cell proliferation was measured after 72 h by CFSE dilution using flow cytometry (BD FACSymphony™ A3, BD Bioscience, San Jose, CA, USA). The levels of IFNγ and IL-2 in the co-cultured (T cells: BM-MDSCs) supernatant were quantified using ELISA kits (Ray Biotech, Peachtree Corners, GA, USA) according to the manufacturer’s instructions.

### 2.7. In Vivo Experiments

Female FVB mice aged 6–8-week-old were purchased from Jackson Laboratory (Bar Harbor, ME, USA). All animal experiments were approved by the Institutional Animal Care and Use Committee (IACUC) of Louisiana State University Health Sciences Center New Orleans (LSUHSC-NO). Mice were housed at 22 °C under a 12 h light/dark cycle with free access to standard diet and water. After an acclimation period, one million C0321 cells were implanted into the fourth mammary fat pad. One week later, when tumors became palpable, mice were treated daily with vehicle or ADT-030 by oral gavage (50 mg/kg body weight). For combined therapy experiments, tumor-bearing mice were treated daily with vehicle or ADT-030 (50 mg/kg body weight) alone or in combination with paclitaxel (10 mg/kg body weight, intraperitoneally, twice weekly). Body weight and tumor volume were monitored twice a week. After three weeks of treatment, mice were euthanized by carbon dioxide (CO_2_) inhalation followed by thoracotomy incision and heart removal. Blood and tumor tissues were collected immediately for further analysis.

### 2.8. RNA Sequencing and Data Analysis

Recently, we described the immunogenic diversity of TNBC patients in obese and non-obese black and white women [22]. Within the scope of the project, we collected 253 TNBC patient samples in collaboration with the Louisiana Tumor Registry (LTR) and completed RNA sequencing (RNA-seq). Using the same RNA-seq data, we completed a secondary data analysis to evaluate PDE10A expression across all TNBC subtypes.

For mouse tumor tissues, total RNA was extracted from fresh frozen tissue samples using the RNeasy mini kit (Qiagen, Germantown, MD, USA). Directions from the manufacturer were followed. Frozen tissues (30–50 mg) were first disrupted and homogenized by bead-milling using 2.8mm ceramic beads (Fisher Scientific, Waltham, MA, USA) and the Omni Bead Ruptor 4 (Omni International, Kennesaw, GA, USA) followed by full-speed centrifugation. After homogenization, the supernatant was purified using RNA purification columns as per the manufacturer’s instructions. A DNase I treatment (Qiagen, Germantown, MD, USA) was included. RNA was eluted in 30 μL of nuclease-free H_2_O after 1 min of incubation at room temperature. RNA quantification was performed using the Qubit RNA Broad Range Assay kit (Cat. #Q33231, Invitrogen, Waltham, MA, USA), and RNA quality was assessed with the Agilent 2100 Bioanalyzer (Agilent Technologies, Santa Clara, CA, USA). Libraries were generated using Illumina Stranded Total RNA prep with Ribo-Zero Plus library preparation kit (cat# 20040526, Illumina, San Diego, CA, USA) according to manufacturer’s instructions. Briefly, ribosomal RNA was first depleted from total RNA (100 ng). Following purification, the RNA was fragmented, and first- and second-strand cDNA was synthesized. Libraries were created from the cDNA by first adenylating the 3′ end and ligating anchors to the ends. An amplification step of 13 cycles of PCR was then performed to add unique indexes to the ligated products. Resulting libraries were quantified using the Qubit dsDNA High Sensitivity Assay Kit (Cat. #Q33231, Invitrogen, Waltham, MA, USA), and size and purity were assessed with the Agilent 2100 Bioanalyzer (Agilent Technologies, Santa Clara, CA, USA). Sequencing was performed on Illumina’s NextSeq 2000 instrument (Illumina, San Diego, CA, USA) using a NextSeq 2000 P2 200 cycles kit (Cat. #20100981, Illumina, San Diego, CA, USA) with pair-end 76 bp reads.

FASTQ files were uploaded to Partek Flow (v12.9.1), and contaminant reads (rDNA, tRNA, mtDNA) were removed using Bowtie 2 (v2.2.5). Based on the quality, the first 35 bp were trimmed, and reads were aligned to STAR v2.7.8a. Quantification was performed against mm 10, and features with ≤10 reads were filtered out and not considered for downstream analysis. Two normalizations were done: (1) TMM normalization (+0.0001 − TMM − log2); (2) TMM normalization (+0.0001 − TMM − log2), followed by baseline normalization to remove the effect of the reference group (subtracting the median expression from each gene in the control group from the treatment group). Comparisons against the reference group were performed using Normalization #1; comparisons between treatments were performed using Normalization #2. Heatmaps and volcano plots were generated, even though the volcano plots were generated only for data obtained from Normalization #2, because in the analysis against the control, none of the genes identified passed multiple corrections (FDR ≤ 0.05). Pathways were generated using KEGG.

### 2.9. Quantitative Real-Time PCR (qPCR)

Total RNA was extracted from fresh frozen tissue samples using the RNeasy mini kit (Qiagen, Germantown, MD, USA). RNA was converted into cDNA using iScript Reverse Transcription Supermix for RT-PCR (Bio-Rad, Hercules, CA, USA). qPCR was carried out using QuantStudio 12K Flex Real-Time PCR System (Applied Biosystems, Waltham, MA, USA) with PowerTrack™ SYBR Green Master Mix (Applied Biosystems, Waltham, MA, USA). The results were normalized to β-actin as a housekeeping gene. Gene expression was determined using the following formula: 2^−ΔΔCT^, where ΔΔCT = ΔCT (treated sample) − ΔCT (control sample).

### 2.10. Kaplan–Meier (KM) Analysis

The statistical method, KM analysis, is used to estimate the probability of survival, disease relapse, or the probability of an event over time. We performed KM plotter analysis [23] by selecting gene chips, breast cancer, and gene symbol *PDE10A (205501_at)*. Split patients’ type was selected as median with relapse-free survival (RFS). We restricted the analysis to subtypes ER (ER status: negative), PR (PR status: negative), and HER2 (HER2 status: negative) to obtain the graph. The KM plotter determined higher and lower PDE10A expression using the median expression values as the default cutoff.

### 2.11. Statistical Analysis

One-way ANOVA was performed to determine the significant differences among the groups. The significance level was considered when *p* ≤ 0.05. GraphPad Prism (version 10.6.0) was used for graphing and data analysis.

## 3. Results

### 3.1. PDE10 Expression Was Markedly Elevated in TNBC

**Figure 1:**
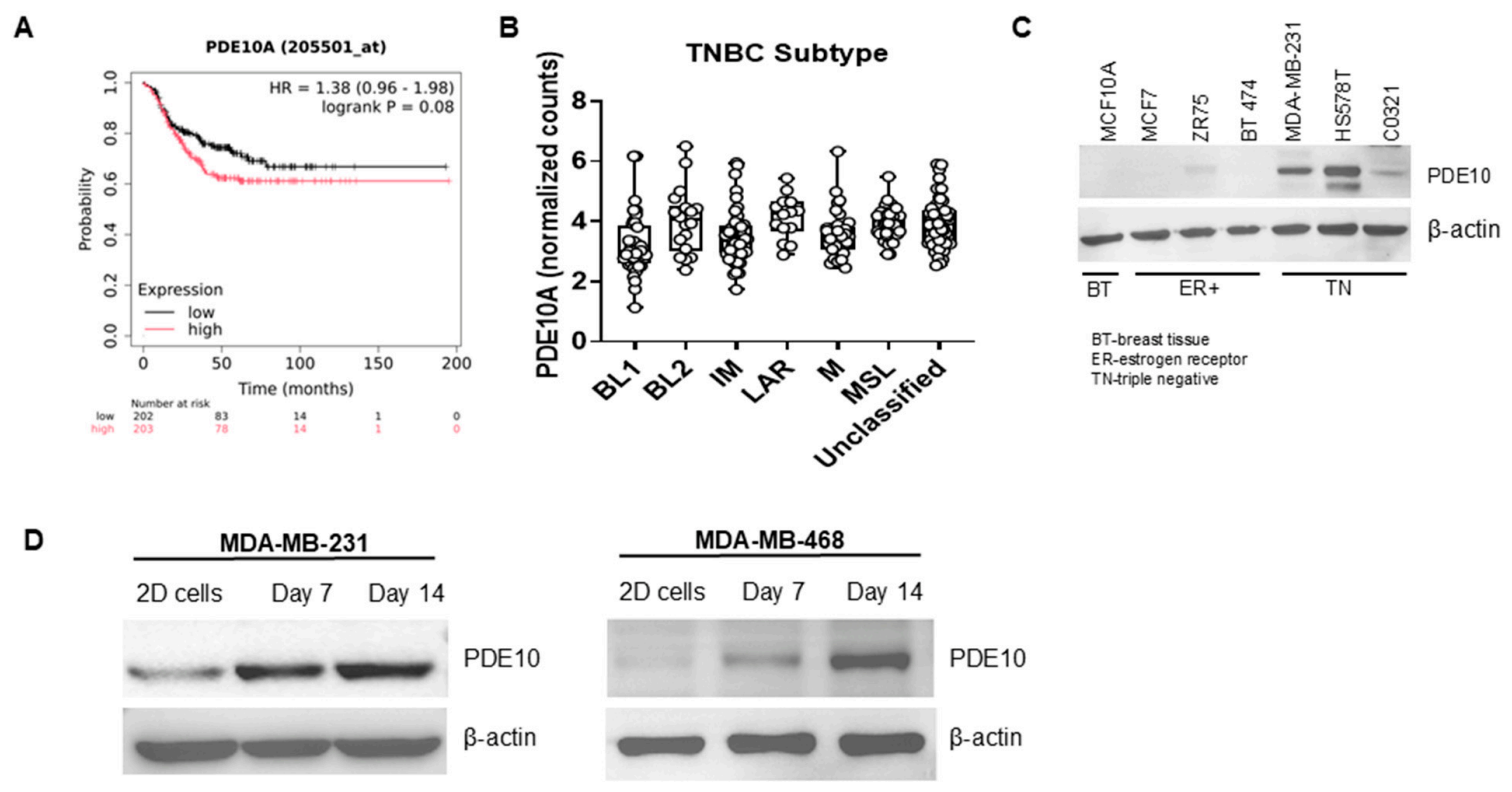
PDE10 was highly expressed in TNBC. (**A**) Kaplan–Meier analysis showed an association between PDE10 expression and overall survival of TNBC patients. Patients with higher *PDE10* expressions had shorter overall survival compared with those with lower expression levels. (**B**) Analysis of RNA-seq data from 253 TNBC patients showed *PDE10* expression across all six TNBC subtypes: BL1, BL2, IM, LAR, M, MSL, and unclassified. (**C**) Levels of PDE10 in normal human mammary epithelial cells (MCF10A), ER+ BC, and TNBC cell lines. (**D**) MDA-MB-231 and MDA-MB-468 cells were cultured for mammospheres formation in MammoCult media. Primary mammospheres were collected on day 7 (P0), and P1 mammospheres were cultured for 7 and 14 days. Mammospheres were collected and prepared for WB. Samples were analyzed by immunoblotting with rabbit monoclonal anti-PDE10A or mouse monoclonal anti-β-actin antibody under reducing conditions, followed by incubation with HRP-conjugated anti-rabbit IgG or anti-mouse IgG. All data were analyzed by the Chemidoc instrument.

The 5-year event-free survival after surgery of stage II–III TNBC patients was approximately 70.3% [24]. Using Kaplan–Meier (KM) analysis [25], we found that PDE10 expression was associated with poorer relapse-free survival in TNBC patients. Patients with higher *PDE10* expression had shorter overall survival than those with lower expression (Figure 1A).

Previously, we analyzed the immunogenomic diversity in 253 TNBC patient samples from different races and obesity status [22]. We used the algorithm described previously to clarify the TNBC subtypes, namely basal-like1 (BL1), basal-like-2 (BL2), immunomodulatory (IM), luminal androgen receptor (LAR), mesenchymal (M), and mesenchymal stem-like (MSL), based on gene expression patterns [26,27]. From the same samples, we further analyzed *PDE10* expression and found it was expressed in all six TNBC subtypes (Figure 1B). Since we observed *PDE10* expression in patients’ samples, we confirmed its expression in human TNBC cell lines. TNBC cells had higher levels of PDE10 compared to ER-positive breast cancer cell lines, as well as normal mammary epithelial cells (Figure 1C). We also analyzed PDE10 levels in TNBC mammospheres derived from MDA-MB-231 and MDA-MB-468 cell lines. Mammospheres generated as described under Methods and Materials were collected after days 7 and 14 and analyzed for PDE10 levels by WB. Our results indicated that PDE10 levels on days 7 and 14 were higher than in 2D culture (Figure 1D). Together, these results suggest that elevated PDE10 levels in TNBC could play a role in the development or progression of TNBC and serve as a potential biomarker for TNBC detection.

### 3.2. ADT-030 Inhibited Cell Growth and Reduced Colony-Forming Capacity of TNBC Cells

**Figure 2:**
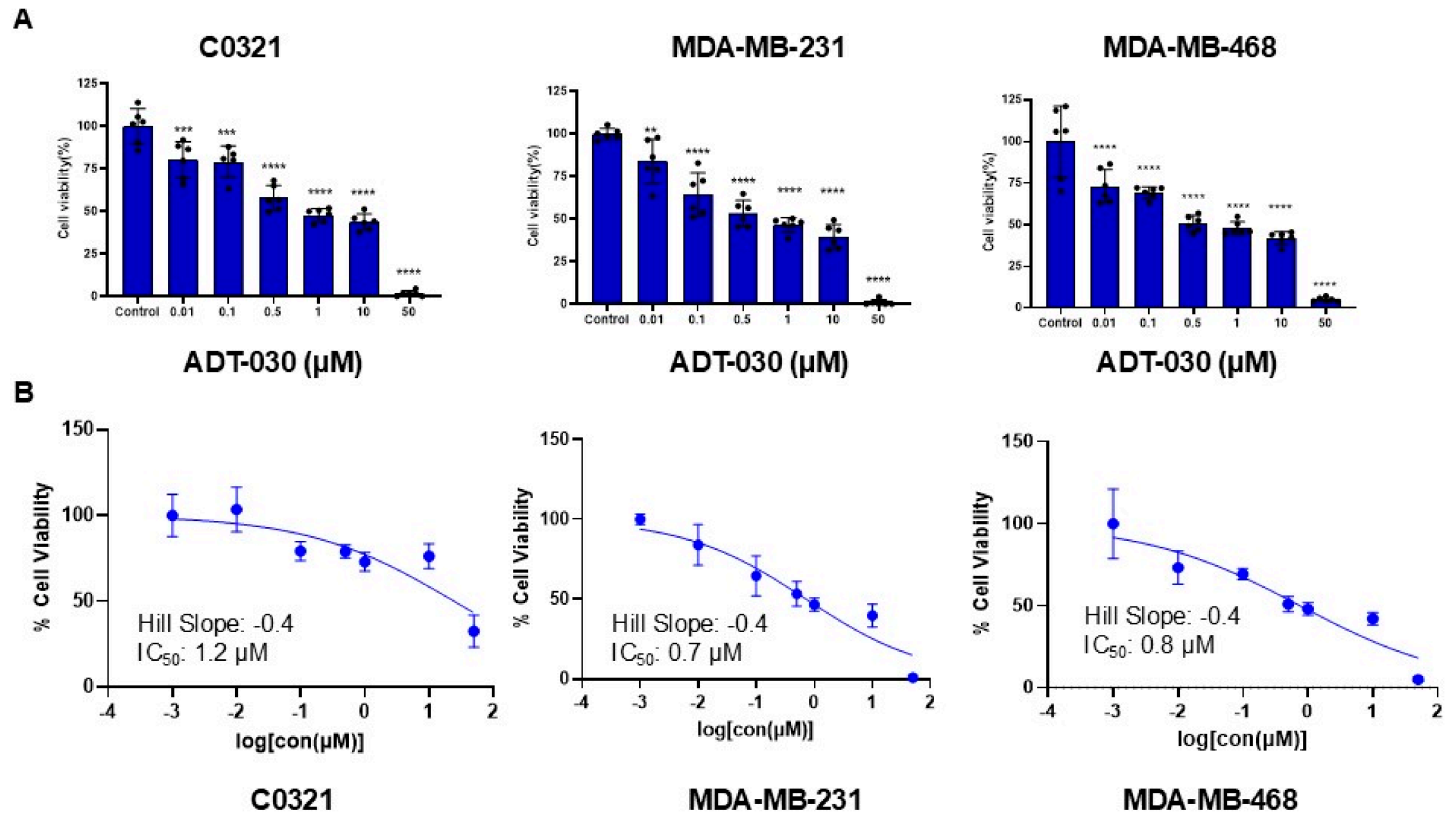
ADT-030 inhibited the proliferation of TNBC cells. (**A**) Cell viability was assessed by MTT assay after treatment with ADT-030. MDA-MB-231, MDA-MB-468, and mouse C0321 cells were treated with 0, 0.01, 0.1, 0.5, 1, 10, and 50 µM of ADT-030 for 48 h. ADT-030 significantly reduced TNBC cell proliferation in a dose-dependent manner. (**B**) IC_50_ was calculated using GraphPad Prism by selecting log (inhibitor) vs. normalized response-variable slope. Data are presented as mean ± SD, and a *p*-value ≤ 0.05 is considered statistically significant. **, *p* < 0.01; ***, *p* < 0.001; ****, *p* < 0.0001.

Previous studies evaluating the oncogenic role of PDE10 have reported expression in colon, ovarian, and lung cancers but low expression in normal tissues outside the CNS, where there is no known physiological function [13,14,15]. PDE10 regulates cGMP/PKG and cAMP/PKA signaling, whereby inhibitors were reported to suppress both the Wnt/β-catenin and RAS-dependent MAPK/AKT signaling pathways that are well-known drivers of cancer cell proliferation, survival, and stem cell-like properties [13,14,15,28]. Piazza and his colleagues have developed and characterized a novel PDE10 inhibitor, ADT-030 [17,18]. We treated human TNBC cells (MDA-MB-231 and MDA-MB-468) and a mouse TNBC cell line (C0321) with different concentrations of ADT-030 and determined its effect on cell viability using the MTT assay. Our results demonstrated that ADT-030 significantly decreased cell viability in a concentration-dependent manner (Figure 2A) with IC_50_ values for all cell lines ranging from approximately 0.7 to 1.2 µM. (Figure 2B). We also analyzed the effect of ADT-030 on normal breast epithelial cells, MCF10A, and our results indicated that the higher dose of ADT-030 can inhibit MCF10A cell proliferation with an IC_50_ 24.4 µM (Figure S1A,B). The effects of ADT-030 on the colony-forming ability of TNBC cells were identified by treating the cells with different concentrations of ADT-030. The results revealed that ADT-030 markedly inhibited colony formation (Figure S1C). Collectively, our results demonstrate that ADT-030 can suppress TNBC cell growth in vitro.

### 3.3. ADT-030 Exhibited Antitumor Activity in Syngeneic TNBC Mouse Model

**Figure 3:**
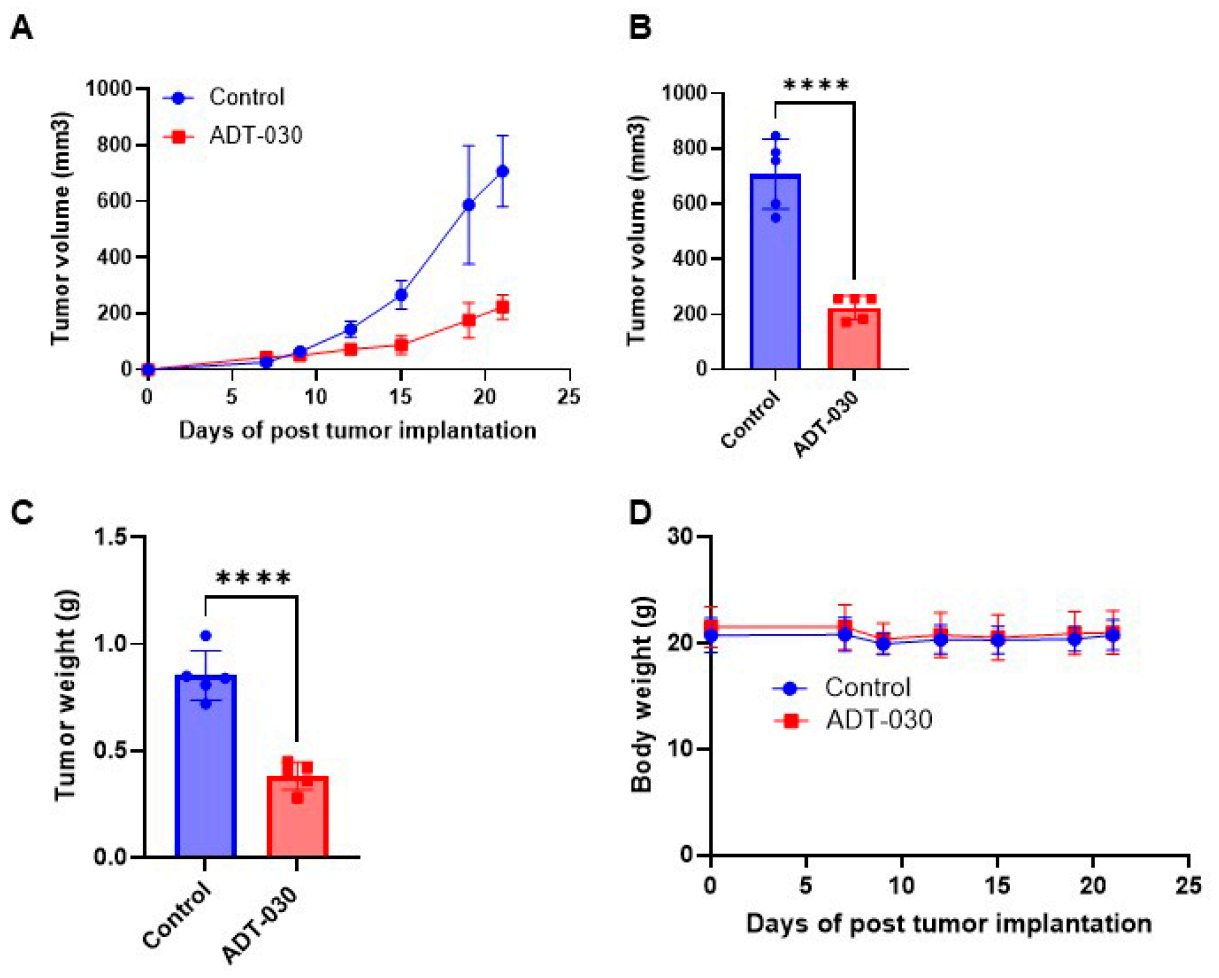
ADT-030 suppressed the syngeneic TNBC tumor growth. Mouse TNBC C0321 cells (1 million) were injected into the 4th mammary fat pads of syngeneic immunocompetent FVB (female) mice. Tumor-bearing mice were treated with vehicle or ADT-030 (50 mg/kg body weight, orally) starting one week after tumor cells inoculation and continued for another two weeks. Tumor volumes and body weights were measured twice a week, and three weeks after tumor cells inoculation; tumors were harvested, weighed, and analyzed. (**A**) Tumor growth, (**B**) tumor volume on day 21, (**C**) tumor weight, and (**D**) mice body weight. Data are presented as mean ± SD, and a *p*-value ≤ 0.05 is considered statistically significant. ****, *p* < 0.0001.

Next, we evaluated the effects of ADT-030 on tumor growth using the C0321, a syngeneic TNBC mouse model as described in our previous study [20]. To test the antitumor efficacy of ADT-030 in the TNBC mouse model, we treated TNBC tumor-bearing mice orally with vehicle or ADT-030 (50 mg/kg body weight) for two weeks. We monitored tumor growth and body weight twice a week. Treatment with ADT-030 resulted in a significant suppression of tumor growth compared with vehicle-treated controls (Figure 3A,B).

On day 21, we measured tumor weight, and the results showed that ADT-030 significantly reduced tumor weight (Figure 3C). No significant body weight loss (Figure 3D) or adverse effects on physical activity attributable to drug toxicity were observed in the ADT-030-treated group, indicating that ADT-030 was well tolerated. Taken together, our results suggest that ADT-030 is a promising well-tolerated drug development candidate for treating TNBC.

### 3.4. ADT-030 Did Not Show Cytotoxic Effects on Immune Cells

**Figure 4:**
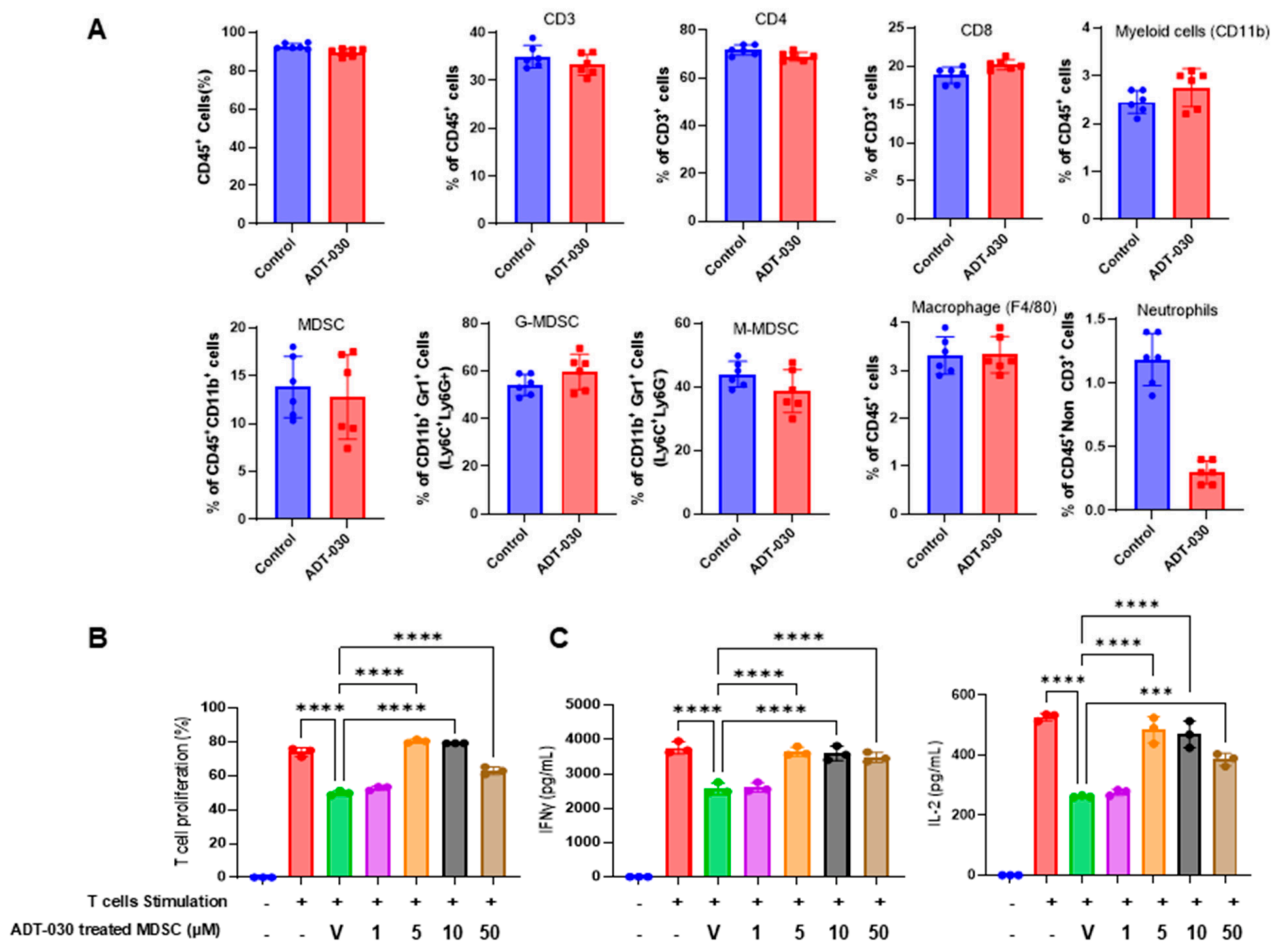
ADT-030 did not affect the immune cells. (**A**) Wild-type female FVB mice were treated daily with vehicle or ADT-030 (50 mg/kg body weight, orally) for 7 days. The spleen was collected, and 1M cells were stained with fluorochrome-conjugated antibodies for 1 h. Cells were washed with PBS, and immune cells population was analyzed by flow cytometry. (**B**) Mouse bone marrow cells were treated with DMSO (V) or different concentrations of ADT-030 (1, 5, 10, 50 µM) to generate BM-MDSC as per the methods described in the Section 2. After 96 h of treatment, BM-MDSCs were collected. T cells from naive mouse spleen were collected, labeled with CFSE, and co-cultured with ADT-030-treated BM-MDSC in the ratio of 4:1 (1M T cells: 0.25M ADT-030-treated MDSC) in a CD3/CD28 (1 µg/mL)-coated 24-well plate. After 72 h of culture, cells were analyzed by flow cytometry, and supernatants were collected. (**C**) The levels of IFNγ and IL-2 in the supernatant were quantified using ELISA kits according to the manufacturer’s instructions. Data are presented as mean ± SD, and a *p*-value ≤ 0.05 is considered statistically significant. ***, *p* < 0.001; ****, *p* < 0.0001.

Developing a new anticancer drug can be challenging due to systemic toxicity. In our present study, we evaluated the safety of ADT-030 in FVB wild-type mice by treating them with ADT-030 (50 mg/kg body weight, orally) for one week. We monitored body weight and, after one week, we collected spleen and blood for further analysis. ADT-030 treatment did not change the mouse’s body weight (Figure S2A). We stained spleen and blood cells with fluorescence-labeled antibodies and analyzed them by flow cytometry. The results showed that ADT-030 did not affect immune cell populations in the spleen except neutrophils (Figure 4A), or in blood (Supplementary Figure S2B). Next, we evaluated the effect of ADT-030 on myeloid-derived suppressor cells (MDSCs), which are among the major immunosuppressive cells in the TME [29]. We examined the effect of ADT-030 on bone marrow-derived MDSC (BM-MDSC)’s inhibitory activities on T cell proliferation. BM-MDSC significantly suppressed T cell proliferation as expected (Figure 4B). We found that ADT-030 treatment (5 µM or above) significantly suppressed the inhibitory activity of BM-MDSCs on T cell proliferation (Figure 4B). Additionally, we measured the levels of IFNγ and IL-2, drivers of T cell proliferation, in the cell culture medium. BM-MDSC significantly reduced IFNγ and IL-2 levels, as expected (Figure 4C). Importantly, ADT-030-pretreated BM-MDSC (5 µM or above) increased IFNγ and IL-2 levels (Figure 4C). These findings indicate that ADT-030 significantly suppressed the inhibitory activities of MDSC on T cell proliferation. Together, our findings provide evidence that ADT-030 is not toxic to immune cells, including T cells, and may possess anti-MDSC activity.

### 3.5. ADT-030 Enhanced Efficacy of Chemotherapy In Vitro and In Vivo

**Figure 5:**
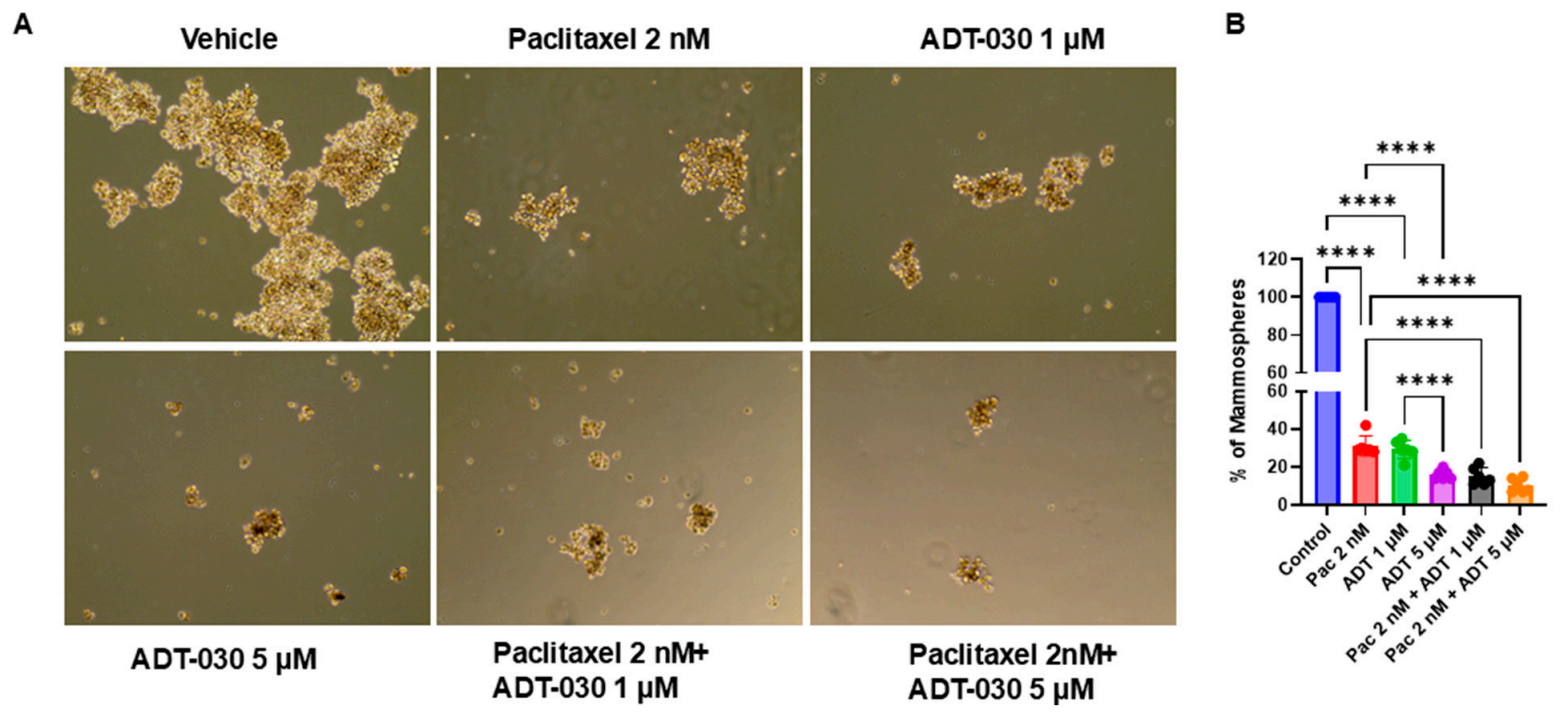
ADT-030 inhibited mammosphere formation. MDA-MB-468 cells were cultured in a low-adhesion plate for mammosphere formation in MammoCult media. P1 (on day 7) mammospheres were treated with vehicle (DMSO) or 1 and 5 µM of ADT-030 alone or in combination with paclitaxel (2 nM). After the treatment, on day 7, mammospheres were counted. (**A**) Representative images from each condition. (**B**) Graphical representation of mammospheres percentages for each condition. Data are presented as mean ± SD, and a *p*-value ≤ 0.05 is considered statistically significant. ****, *p* < 0.0001.

**Figure 6:**
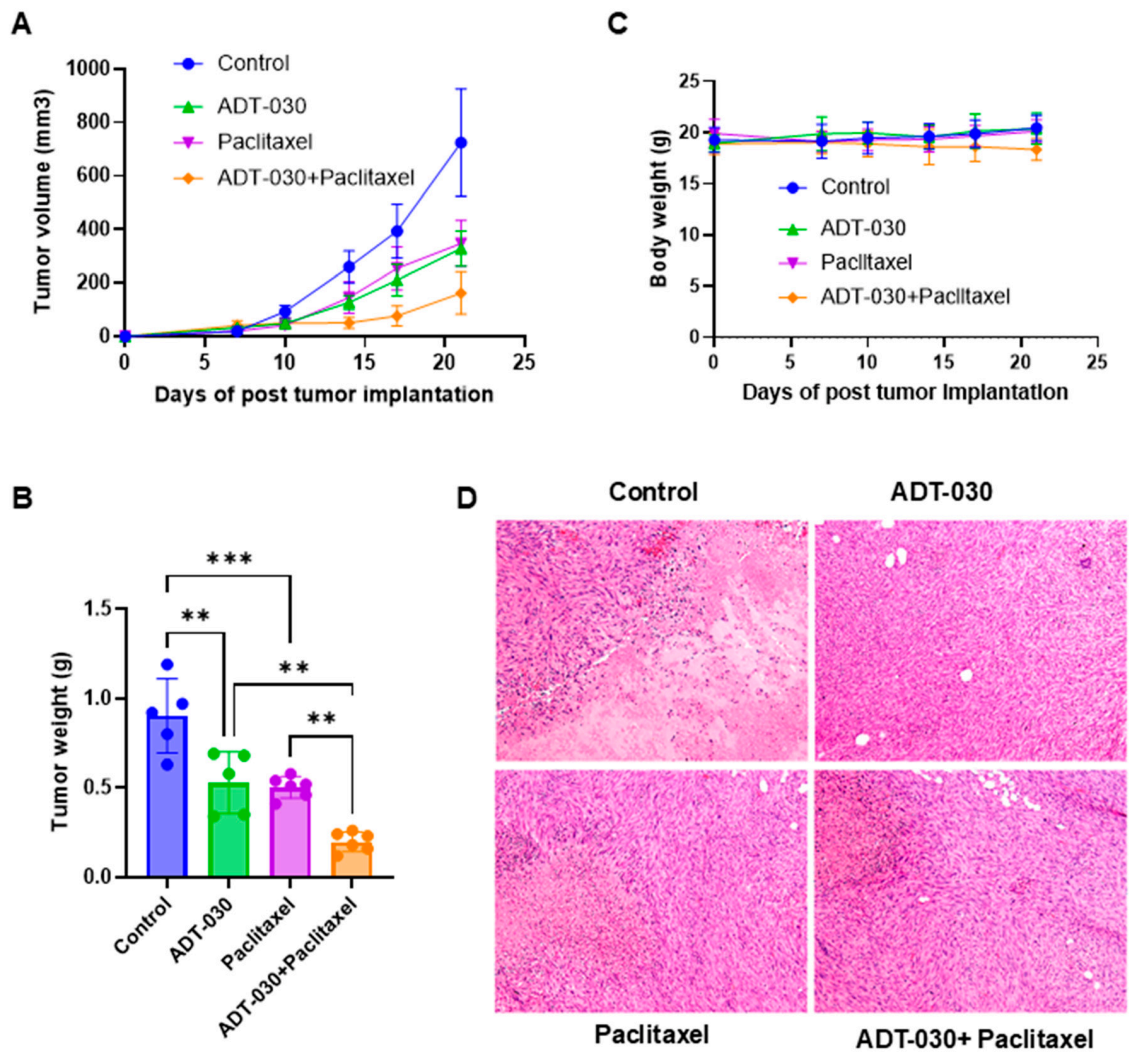
ADT-030 potentiated the efficacy of chemotherapy on syngeneic TNBC tumor growth. Mouse TNBC C0321 cells (1 million) were injected into the 4th mammary fat pads of syngeneic immunocompetent female FVB mice. Tumor-bearing mice were treated daily with vehicle or ADT-030 (50 mg/kg body weight, orally), paclitaxel (10 mg/kg body weight, twice a week, ip), or both, starting one week after tumor cell inoculation for two weeks. Tumor volumes and body weights were measured twice per week, and three weeks after tumor cell inoculation, tumors were harvested, weighed, and analyzed. (**A**) Tumor growth, (**B**) tumor weight, (**C**) mice body weight, and (**D**) representative H & E staining of collected tumors. Data are presented as mean ± SD, and a *p*-value ≤ 0.05 is considered statistically significant. **, *p* < 0.01; ***, *p* < 0.001.

After confirming ADT-030’s tumor growth inhibitory activity, we focused on analyzing its effects on the self-renewal capacity of cancer stem-like cells (CSCs). One of the major drawbacks for cancer drug development is the presence of self-renewing CSCs that contribute to disease relapses and metastasis [30,31,32]. To investigate whether ADT-030 inhibits the self-renewal capacity of CSCs, we treated mammospheres (an in vitro tumor model of CSCs) with ADT-030 alone or in combination with paclitaxel. The results indicated that ADT-030 alone or in combination with paclitaxel significantly inhibited mammosphere formation of MDA-MB-468 (Figure 5A,B) and MDA-MB-231 cells (Figure S3A,B). These results encouraged us to use this combination in a TNBC syngeneic mouse model. We treated TNBC tumor-bearing mice with vehicle or ADT-030 (50 mg/kg body weight, orally, once daily), either alone or in combination with paclitaxel (10 mg/kg body weight, intraperitoneally, twice weekly). Although both ADT-030 and paclitaxel alone significantly suppressed the TNBC tumor growth, the greatest suppression was observed in the combined treatment group (Figure 6A,B) without significant effects on body weight (Figure 6C). Hematoxylin/Eosin (H & E) staining was performed to check the morphology of tumor tissues, which confirmed that ADT-030 treatment reduced tumor burden (Figure 6D). Together, these results confirmed the antitumor activity of ADT-030 as a monotherapy and demonstrate that ADT-030 enhanced the antitumor efficacy of chemotherapy both in vitro and in vivo.

### 3.6. ADT-030 Treatments Modulated Cellular Processes and Signaling Pathways

**Figure 7:**
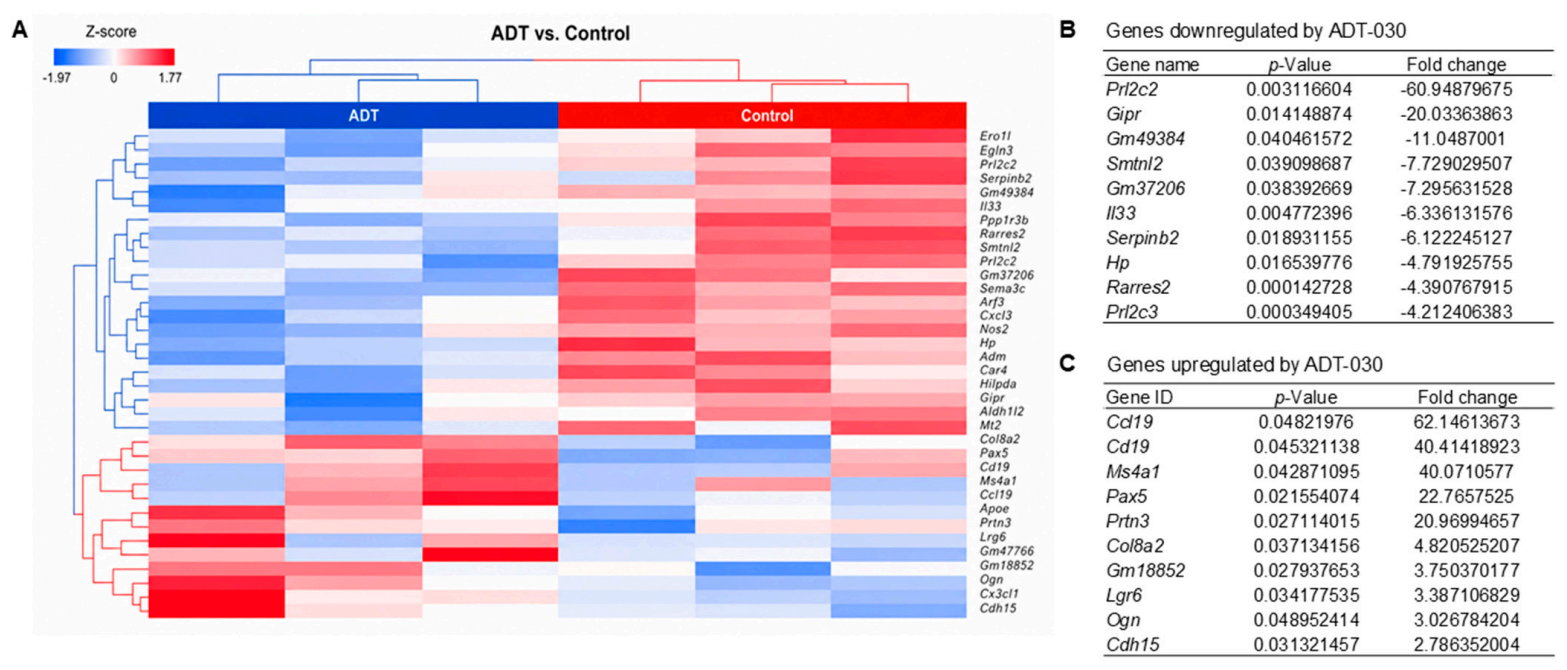
Differentially expressed genes were significantly altered. We performed RNA-seq using tumor tissues from the experiment described in Figure 6. (**A**) Hierarchical clustering of expression changes seen by RNA-seq between ADT-030-treated and control groups. Expression values were presented as z-scores, with red representing higher and blue representing lower expression. Top 10 downregulated (**B**) and upregulated (**C**) genes by ADT-030 treatment.

We performed RNA-seq on tumor samples to elucidate the mechanism by which ADT-030 enhanced antitumor efficacy, either alone or in combination with paclitaxel. We explored the target genes, pathways, and gene ontologies involved in the tumor-suppressing activities of ADT-030. It has been reported that genes, cellular processes, and signaling pathways play a significant role in regulating tumor initiation, growth, and progression [33,34]. We identified DEGsthat were significantly (*p* ≤ 0.05) modulated by ADT-030 (Figure 7A).

Within the control vs ADT-030-treated group, prolactin-2C2 (*Prl2c2*), gastric inhibitory polypeptide receptor (*Gipr*), smoothelin-like 2 (*Smtnl2*), and other genes were significantly downregulated, whereas *Ccl19*, *Cd19*, *Ms4a1*, and other genes were significantly upregulated. Among those genes, the top 10 downregulated and upregulated genes significantly modulated by ADT-030 are listed (Figure 7B,C). Further, we cross-validated the expression of some genes by qPCR and obtained similar tendencies of down- and up-regulation of these genes as obtained by DEGs (Figure S4). We investigated the signaling pathways modified by ADT-030 treatment. We found that the NF-kappa B (NF-κB) signaling pathway, cytokine–cytokine receptor interaction, TNF signaling pathway, and HIF-1 signaling pathway were among the top 10 pathways affected by ADT-030 treatment (Table 1).

Gene ontology (GO) identified cellular processes, including extracellular space, extracellular region part, and extracellular region, among the top 10 cellular processes significantly modulated by ADT-030, as listed in Table 2.

We also analyzed the DEGs, pathways, and GO between the control and combination treatment groups. Combined treatment of ADT-030 with paclitaxel significantly altered the expression of DEGs. Functional enrichment analysis revealed that these genes were associated with cellular processes, including regulation of cell motility. The top 10 downregulated or upregulated genes, enriched signaling pathways, and GO were listed in Supplemental Figure S4.

## 4. Discussion

TNBC is one of the most aggressive and deadliest forms of breast cancer with a high mortality rate due to therapy resistance and disease recurrence [35]. Treatment of TNBC solely relies on chemotherapy, in some cases with immunotherapy. Although several target-directed drugs and immune checkpoint inhibitors are used, including olaparib, talazoparib, atezolizumab, sacituzumab-govitecan, and pembrolizumab [36]. Due to limited therapeutic options, new targeted strategies are urgently needed for TNBC treatment.

KM analysis showed that elevated levels of PDE10 are associated with poor relapse-free survival among TNBC patients. Our results demonstrate that *PDE10* is expressed in all TNBC subtypes. Based on previous studies, PDE10 is a promising target for various cancers [13,14,15]. We therefore considered PDE10 as a candidate therapeutic target for TNBC. ADT-030, a novel PDE10 inhibitor, has been developed and characterized by Piazza and his colleagues. ADT-030 showed robust and durable antitumor and antimetastatic activity in various cancer models, including PDAC [17,18]. In our current study, we evaluated the role of PDE10 in TNBC using ADT-030 and a selective inhibitor. Our results indicated that ADT-030 significantly inhibited TNBC cell proliferation. A recent study demonstrated that ADT-030 inhibited the clonogenic abilities of PDAC cells and in vivo PDAC tumor growth [18]. To evaluate the efficacy of ADT-030 using TNBC mouse tumor models, we treated TNBC tumor-bearing mice with ADT-030 and observed significant suppression of tumor growth without adverse effects.

Developing a new drug can be challenging due to systemic toxicities. A recent study concluded that ADT-030 showed a favorable toxicity profile in mice [17]. In our current study, we also observed that treatment with ADT-030 did not result in visual abnormalities, signs of toxicity, weight loss, diarrhea, or behavioral changes in mice. ADT-030 also did not adversely affect the normal immune cell populations that we studied, including MDSCs, T cells, myeloid cells, and macrophages, except neutrophils. A previous study revealed that PDE10 inhibition increased intracellular cAMP/cGMP levels [14], which ultimately activated PKA/PKG signaling pathways that suppressed NF-κB-dependent inflammatory cytokines or growth factors required for neutrophil production and survival [37]. This may be a mechanistic way to downregulate neutrophils in the spleen. However, further studies are necessary to prove this mechanism. We also analyzed the effect of ADT-030 on BM-MDSC’s inhibitory activities on T cell proliferation. Our results revealed that ADT-030 inhibited MDSC-mediated suppression of T cell proliferation without adversely affecting normal T cells.

We also tested the efficacy of ADT-030 in combination with chemotherapy. It is well established that monotherapy is seldom sufficient to treat cancer and that combination therapy offers greater efficacy [38]. Previous reports suggest that different drug combinations enhance the therapeutic efficacy of cancer treatment [39,40]. In the present study, we found that ADT-030 enhanced the efficacy of paclitaxel to inhibit mammosphere growth in vitro. The in vitro chemotherapy-enhancing activity of ADT-030 prompted us to evaluate its combined effects in a TNBC mouse model. Our results indicated that ADT-030 alone significantly suppressed tumor growth and, notably, potentiated the therapeutic efficacy of paclitaxel, with the highest suppression observed in the combination.

The mechanism of action of PDE10 inhibitors in colon, ovarian, and lung cancers is summarized in a review [41]. It has been reported that in colon, lung, and ovarian cancer cell lines, inhibition of PDE10 with known inhibitors suppresses Wnt-induced β-catenin signaling as well as RAS-mediated MAPK/AKT oncogenic pathways by activating cGMP/PKG and/or cAMP/PKA signaling [13,14,15]. Recent studies revealed that ADT-030 inhibited PDAC tumor growth by a similar mechanism involving dual suppression of RAS/β-catenin signaling pathways [17,18]. In the present study, we investigated the mechanism by which ADT-030 suppresses tumor growth, either alone or in combination with paclitaxel in TNBC. RNA-seq analysis revealed that DEGs were significantly altered by ADT-030 treatment. *Prl2c2* is one of the genes downregulated by ADT-030. A previous study reported that *Prl2c2* enhanced tumorigenesis in ovarian cancer [42]. Another downregulated gene was *Gipr*, which is a metabolic regulator but possesses a role in various cancers, including breast, colorectal, endometrial, lung, pancreatic, and renal cancers [43]. Smtnl2 is an actin-binding protein that contributes to cytoskeletal organization and acts as a tumor suppressor. *Smtnl2* was downregulated in kidney and breast cancers compared to normal tissues; the absence of *Smntl2* promoted cancer-related actin dynamics [44]. We observed that *Smtnl2* was downregulated by ADT-030 treatment, consistent with previous findings [44]. Among the upregulated genes, *Ccl19* is a cytokine that guides T cells or dendritic cells to lymphoid organs. However, Ccl19 plays a dual role in cancer, as a tumor suppressor or promoter of metastasis [45]. CD19 is a well-known transmembrane glycoprotein expressed on B cells and is responsible for B cell development, maturation, activation, and tolerance [46]. In the current study, we also found that ADT-030 treatment upregulated *CD19* expression. This result demonstrates that ADT-030 may modulate the TME by activating B cells or by increasing B cell infiltration into tumors, which in humans is associated with better outcomes, as described [47]. However, further studies are warranted to explore the complex mechanisms by which ADT-030 influences this signaling pathway. From our pathway analysis, we observed that ADT-030 treatment upregulated many signaling pathways. Among them, the NF-κB signaling pathway is a key regulator of normal cell homeostasis, while overexpression may promote tumor initiation, cell proliferation, metastasis, and inhibit apoptosis [48,49]. In TNBC, aberrant expression of the NF-κB signaling pathway is reported [50]. However, ADT-030 treatment did not appear to activate this pathway.

Cytokine–cytokine receptor interactions and TNF-α signaling are crucial cell signaling pathways in which cytokines bind to cell-surface receptors to promote immune activation, cell proliferation, and differentiation [51]. In the context of cancer, cytokine–cytokine receptor interactions and TNF-α signaling exhibit dual functions, promoting both protumor and antitumor activity [52,53,54]. A previous study demonstrated upregulation of TNF-α in TNBC [55]. In our study, we also identified an upregulation of the TNF-α signaling pathway. Further studies are necessary to determine whether ADT-030 works through this pathway. ADT-030 modulates many cellular processes, including the extracellular space, which comprises the extracellular matrix and interstitial fluids. Considering the wide range of functions of the extracellular space, it may regulate tumor initiation, progression, angiogenesis, and metastasis [56,57]. The extracellular region, signaling receptor binding, and other cellular processes are also modulated by ADT-030 treatment. Further studies are required to identify the specific mechanisms of action of ADT-030 in cellular processes. Although ADT-030 showed potency against TNBC, we should acknowledge some limitations of this study. Several drug-related factors, such as pharmacokinetics, in vivo application potency, biomarker selection, and other considerations, should be addressed before proceeding to translational applications of this compound. However, we have a strong interest in conducting further experiments to explore the full potencies of this compound.

## 5. Conclusions

Here, for the first time, we described the role of PDE10 in TNBC. We studied the novel PDE10 inhibitor, ADT-030, and concluded that PDE10 is a potential therapeutic target for TNBC, either alone or in combination with paclitaxel. Moreover, our RNA-seq data suggests that ADT-030 treatment influences gene and pathway expression. Furthermore, the current results suggest that ADT-030 may be a drug candidate for translational applications to treat TNBC. However, further studies are warranted to determine the mechanisms by which ADT-030 modulates gene expression and cell-signaling pathways to alter the cellular environment to induce cell death or render tumor cells chemosensitive.

## Figures and Tables

**Table 1:** Pathway enrichment analysis of RNA-seq. Top 10 pathways modulated by ADT-030 are listed in the table.

Description	*p*-Value
NF-kappa B signaling pathway	0.0302084
Cytokine–cytokine receptor interaction	0.00622828
Mineral absorption	0.0098158
Viral protein interaction with cytokine and cytokine receptor	0.00179268
Hematopoietic cell lineage	0.0242283
Nitrogen metabolism	0.0426743
One-carbon pool by folate	0.0475788
TNF signaling pathway	0.0344898
HIF-1 signaling pathway	0.00307583
Amoebiasis	0.030731

**Table 2:** Gene ontology (GO) enrichment analysis of DEGs. Top 10 cellular processes modulated by ADT-030 are listed in the table.

Description	*p*-Value
Extracellular space	1.37 × 10^−13^
Extracellular region part	4.84 × 10^−13^
Extracellular region	5.91 × 10^−10^
Signaling receptor binding	1.43 × 10^−9^
Cellular divalent inorganic cation homeostasis	5.93 × 10^−9^
Receptor ligand activity	9.25 × 10^−9^
Divalent inorganic cation homeostasis	9.76 × 10^−9^
Cation homeostasis	1.14 × 10^−8^
Inorganic ion homeostasis	1.40 × 10^−8^
Receptor regulator activity	1.64 × 10^−8^

## Data Availability

The original contributions presented in this study are included in the article/Supplementary Materials. Further inquiries can be directed at the corresponding author.

## Supplementary Materials

The following supporting information can be downloaded at https://www.mdpi.com/article/10.3390/cells15151374/s1, Figure S1: Effect of ADT-030 on cells growth; Figure S2: ADT-030 did not affect the mouse body weight and immune cells; Figure S3: ADT-030 inhibit mammospheres formation; Figure S4: Differentially expressed genes significantly altered by ADT-030+Paclitaxel treatment; Figure S5: Analysis of genes expression pattern altered by ADT-030 treatment; Figure S6: Original immunoblotting images relative to the expression of PDE10A.
