# Supplementary material for: Developing a Phosphodiesterase 10A Inhibitor as a Novel Therapeutic Agent for Triple-Negative Breast Cancer
Source: Cells. 2026 Jul 30;15(15):1374. doi: 10.3390/cells15151374 (PMC13464762; doi:10.3390/cells15151374)

Developing a Phosphodiesterase 10A Inhibitor as a Novel Therapeutic Target for Triple-Negative Breast Cancer

Mrityunjoy Biswas[1], Md Manirujjaman[1], Jovanny Zabaleta[2], Dorota Wyczechowska[2], Jone Garai[2], Qingzhao Yu[3], Luis Del Valle[2,4], Samarpan Majumder[1], Timothy Kayes[2], Xi Chen[5], Adam B. Keeton[5], Lucio Miele[1,6], Yulia Y. Maxuitenko[5], Nan Li[7], Gary A. Piazza[5] and Fokhrul Hossain[1]*

Figure S1: Effect of ADT-030 on cells growth. (A) Cell viability was assessed by MTT assay after treatment with ADT-030. Human epithelial cells, MCF10A were treated with 0, 0.01, 0.1, 0.5, 1, 10, and 50 µM of ADT-030 for 48 hours. (B) IC_{50} was calculated using GraphPad Prism. (C) MDA-MB-231, MDA-MB-468 and C031cells were treated with vehicle, 10, 100, 500 nM, 1 or 5 µM of ADT-030, cultured for fourteen days, stained with crystal violet, and cell survival was measured using clonogenic assays. ADT-030 treatment decreased the clonogenic potential of TNBC cells. Representative images are shown. Data are presented as mean ± SD, and a P-value ≤0.05 is considered statistically significant. $**p < 0.01$, $***p < 0.001$, and $****p < 0.0001$.

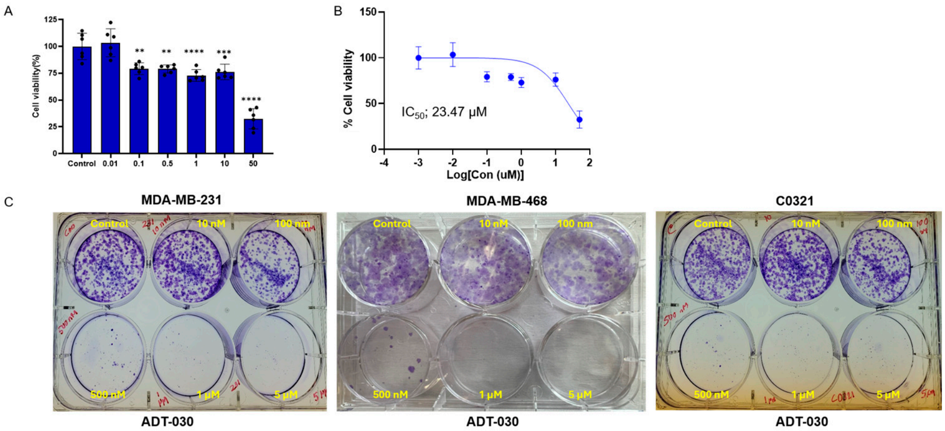

Figure S2: ADT-030 did not affect the mouse body weight and immune cells. Mice were treated daily with vehicle or 50 mg/kg BW of ADT-030 for seven days. (**A**) Body weight. (**B**) Blood cells were collected and stained with fluorescence conjugated antibodies for 1 h and lysed with RBC lysis buffer. Cells were washed with PBS and immune cells population were analyzed by flow cytometry. Data are presented as mean ± SD.

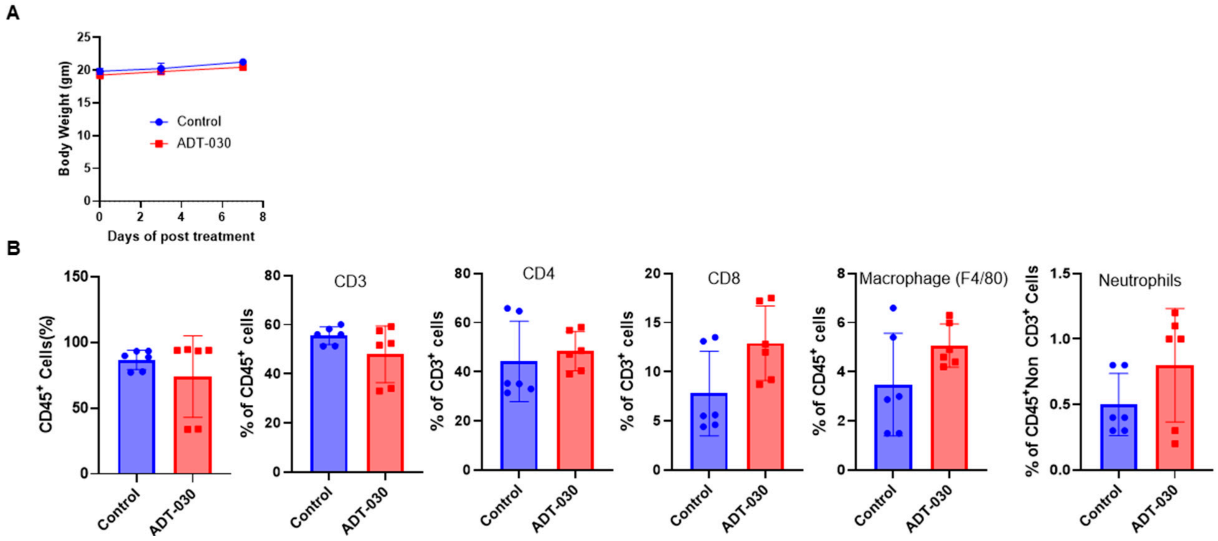

Figure S3: **ADT-030 inhibit mammospheres formation:** MDA-MB-231 cells were cultured in a low-adhesion plate for mammosphere formation in MammoCult media. P1 (on day 7) mammospheres were treated with vehicle (DMSO) or 1 and 5 µM of ADT-030 alone or in combination with paclitaxel (2 nM). After the treatment, on day 7, mammospheres was counted. (**A**) Representative images from each condition. (**B**) Graphical representation of mammospheres percentages for each condition. Data are presented as mean ± SD, and a P-value ≤0.05 is considered statistically significant. ****p < 0.0001.

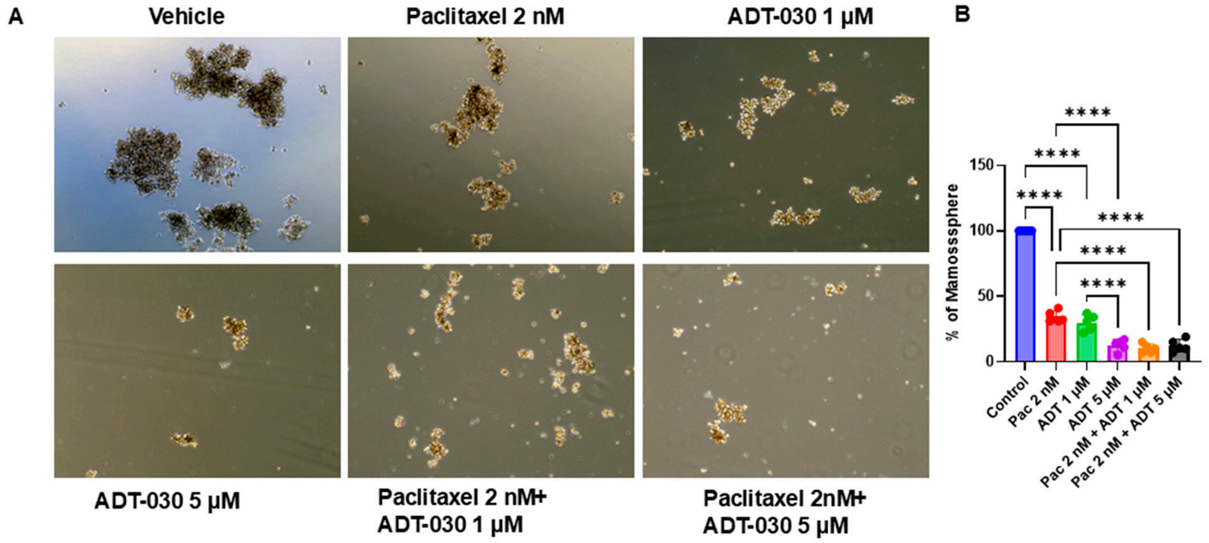

Figure S4: Differentially expressed genes significantly altered by ADT-030+Paclitaxel treatment. We performed RNA-seq using tumor tissues from experiment described in Figure 6. (**A**) Hierarchical clustering of expression changes seen by RNA-seq between control and ADT-030+Paclitaxel. Expression values were presented as z-scores, with red representing higher while blue representing lower expression. Top 10 downregulated (**B**) and upregulated (**C**) genes by ADT-030+Paclitaxel treatment. (**D-E**) Top 10 signaling pathways (**D**) and top 10 cellular processes (**E**) modulated by ADT-030 plus paclitaxel treatment.

A

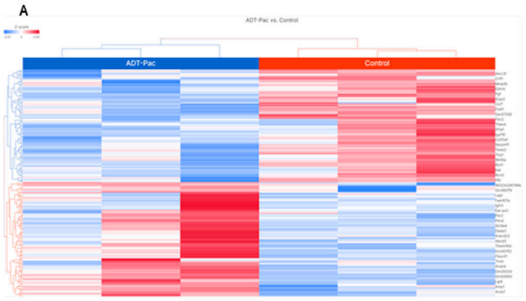

B Genes downregulated by ADT-030+Paclitaxel

Gene name	P value	Fold change
Gm45062	0.010997414	-47.43163234
Gm37206	0.003714439	-34.14972989
Rpl17-ps10	0.00685635	-22.12792211
Thbs4	0.00573742	-13.8029097
Serpinb2	0.014474841	-10.16253441
Xcr1	0.049011611	-9.414308475
Gfra1	0.002784089	-9.154634784
Col23a1	0.008393603	-8.106039946
Mmp13	0.001337746	-7.799558991
Cd209f	0.045174642	-7.569083302

C Genes upregulated by ADT-030+Paclitaxel

Gene name	P value	Fold change
Igfn1	0.021885681	497.2949492
Myh7	0.025922969	417.1491015
Plin1	0.000986951	153.8765823
Chil3	4.63E-05	144.9941569
Aldh1a1	0.005222301	104.2698698
Ankrd2	0.005023569	81.31622095
Abca6	6.51E-05	51.07595612
Zbtb16	0.000525534	50.29999992
4632433K11Rik	0.038984767	37.01053046
Retnla	0.001521377	31.25733973

D

Description	P value
IL-17 signaling pathway	5.71E-07
Viral protein interaction with cytokine and cytokine receptor	6.62E-06
Cytokine-cytokine receptor interaction	5.57E-05
Chemokine signaling pathway	0.000177987
Malaria	0.000282243
Complement and coagulation cascades	0.000409517
TNF signaling pathway	0.00125784
Rheumatoid arthritis	0.002117
ABC transporters	0.00238234
Focal adhesion	0.00571417

E

Description	P value
Regulation of cell migration	1.65E-19
Regulation of cellular component movement	2.84E-19
Regulation of cell motility	1.00E-18
Extracellular region	1.05E-18
Regulation of locomotion	1.37E-17
Extracellular space	1.17E-16
Extracellular matrix	1.29E-16
Extracellular region part	2.66E-16
Regulation of localization	1.85E-15
Positive regulation of cell migration	2.50E-15

Figure S5. Analysis of genes expression pattern altered by ADT-030 treatment. Total RNA was extracted from tumor tissues treated with control and ADT-030. RNA was converted into cDNA and analyzed by quantitative real-time PCR using SYBR Green Master Mix. Relative mRNA expression was normalized to β-actin and quantified using the Ct values. Panel (A) Downregulated genes and (B) Upregulated genes, (C) Primers sequences. Data are expressed as mean ± SD.

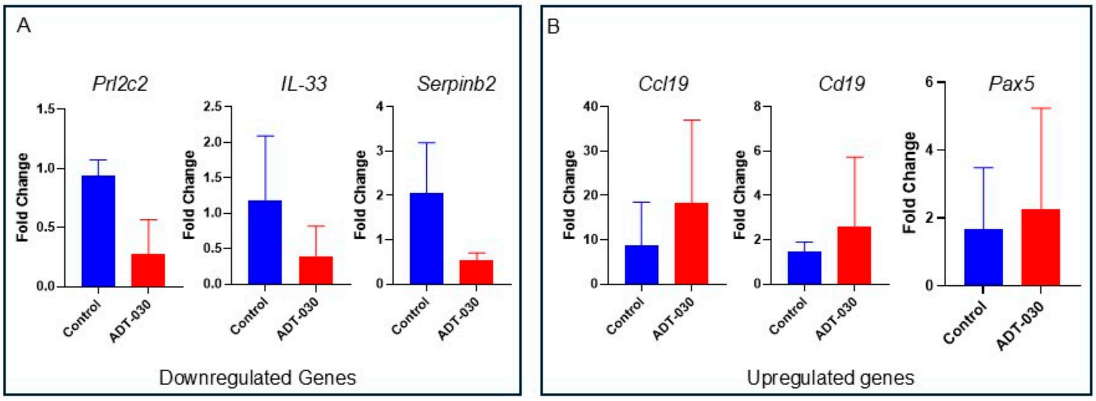

C

Gene Name (Mouse)	Forward Primer (5'-3')	Reverse Primer (5'-3')
Prl2c2	GACTCTGCAGAGATGCTCCC	ATGGTGCTCATGAGGGCATT
IL-33	TCGGCTGCTTGCTTTCCTTA	GTTCCCGGCCTCTTTGATCA
Serpinb2	GGCCATAGTTCTCCTCGGTG	TCATGCGAGTTCACACGGAA
Ccl19	CAGTCACTCCCCTGTGAACC	CTTGGCTGGGTTAGGTCTGG
Cd19	ATGCCATCTCCTCTCCCTGT	GAGGGGCACATACAGGCTTT
Pax5	CATCCCTTTGTCCCTGTCCC	AAGAAGTGAAGGCCCGTGAG
β-actin	GGTGGGGAATGGGTCAGAAG	AGCTCATTGTAGAAGGTGTGG

Figure S6. Original immunoblotting images relative to the expression of PDE10A.(A) TNBC cell lines, the blots relative to figure 1C (in the final figure of manuscript). (B) mammospheres from MDA-MB-231 and MDA-MB-468 cell lines and western blot panels relative to figure 1D (in the final figure of manuscript). Red squares indicate the images used for final figures.

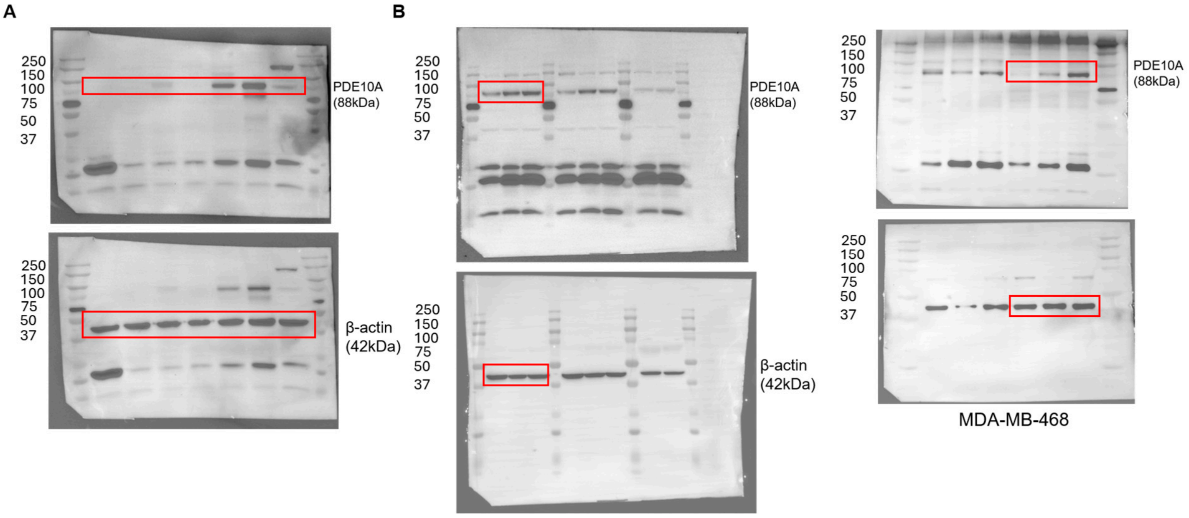

Supplement: Supplementary file 1 [file cells-15-01374-s001.zip › Supplemental figures_ADT-030.pdf]
